# Principal component analysis of physicochemical and sensory characteristics of beef rounds extended with gum arabic from *Acacia senegal* var. *kerensis*

**DOI:** 10.1002/fsn3.576

**Published:** 2018-01-16

**Authors:** Johnson K. Mwove, Lilian A. Gogo, Ben N. Chikamai, Mary Omwamba, Symon M. Mahungu

**Affiliations:** ^1^ Dairy and Food science and Technology Department Egerton University Egerton Kenya; ^2^ Kenya Forest Research Institute Nairobi Kenya

**Keywords:** beef injection, beef round, gum arabic, principal component analysis

## Abstract

Principal component analysis (PCA) was carried out to study the relationship between 24 meat quality measurements taken from beef round samples that were injected with curing brines containing gum arabic (1%, 1.5%, 2%, 2.5%, and 3%) and soy protein concentrate (SPC) (3.5%) at two injection levels (30% and 35%). The measurements used to describe beef round quality were expressible moisture, moisture content, cook yield, possible injection, achieved gum arabic level in beef round, and protein content, as well as descriptive sensory attributes for flavor, texture, basic tastes, feeling factors, color, and overall acceptability. Several significant correlations were found between beef round quality parameters. The highest significant negative and positive correlations were recorded between color intensity and gray color and between color intensity and brown color, respectively. The first seven principal components (PCs) were extracted explaining over 95% of the total variance. The first PC was characterized by texture attributes (hardness and denseness), feeling factors (chemical taste and chemical burn), and two physicochemical properties (expressible moisture and achieved gum arabic level). Taste attribute (saltiness), physicochemical attributes (cook yield and possible injection), and overall acceptability were useful in defining the second PC, while the third PC was characterized by metallic taste, gray color, brown color, and physicochemical attributes (moisture and protein content). The correlation loading plot showed that the distribution of the samples on the axes of the first two PCs allowed for differentiation of samples injected to 30% injection level which were placed on the upper side of the biplot from those injected to 35% which were placed on the lower side. Similarly, beef samples extended with gum arabic and those containing SPC were also visible when scores for the first and third PCs were plotted. Thus, PCA was efficient in analyzing the quality characteristics of beef rounds extended with gum arabic.

## 1 INTRODUCTION

Meat extenders are substances that are added in meat products with the aim of improving the binding properties of such products. This has been achieved through the addition of functional ingredients in the form of inorganic salts (i.e., sodium chloride, phosphates, bicarbonate) and organic compounds from plant and animal origins, such as starch, hydrocolloids, and proteins, to meet wide sensory and technological requirements of processed meat producers and consumers (Petracci, Bianchi, Mudalal, & Cavani, 2013). Extenders such as soy protein concentrates (SPC), whole milk, egg proteins, and fillers such as starches are used for the manufacture of affordable but nutritious meat products (Heinz & Hautzinger, 2007). Studies have shown the effect of some of these nonmeat additives on the quality and physicochemical properties of meat products (Andrès, Zaritzky, & Califano, 2006; Soltanizadeh & Ghiasi‐Esfahani, 2015; Youssef & Barbut, 2011).

Plant‐based hydrocolloids consisting mainly of polysaccharides such as carrageenan (Ayadi, Kechaou, Makni, & Attia, 2009; FAO/WHO, 2007), flaxseed gum, gellan gum (Zhou, Meng, Li, Ma, & Dai, 2010), and gum karaya (Nussinovitch, 2009) have been found to improve water‐holding capacity and appearance of meat products. Recently, for the first time, gum arabic from *Acacia senegal* was reported to enhance cook yield and juiciness when used in extended beef rounds (Mwove, Gogo, Chikamai, Omwamba, & Mahungu, 2016). Conclusions on the quality of these meat and meat products are done based on measurements taken on many quality attributes which are related (Cañeque et al., 2004). According to Karlsson (1992), the large number of measures used to assess meat quality are usually correlated. Therefore, they could be replaced with a few measures while retaining the information. Conclusions based on analysis performed on single physicochemical and sensory characteristics do not provide any indication on the relationships among the various physicochemical and sensory characteristics, nor allow the grouping of samples with similar characteristics. Therefore, there is need to have a few elements to synthesize the trends observed in beef rounds extended with gum arabic and thus draw more information from the large amount of heterogeneous data collected. Moreover, there are minimal reports that elucidate relationships between meat and meat products quality measurements. To achieve this, multivariate statistical methods such as principal component analysis (PCA) can be employed. PCA can be used to extract the important information and reduce a large set of correlated variables to uncorrelated measures, each of which is a particular linear combination of the original quality characteristics, without loss of information (Abdi & Williams, 2010; Karlsson, 1992; Šnirc, Kral, Ošťádalová, Golian, & Tremlová, 2017). The new uncorrelated measures (principal components) equal to the number of the original variables in the heterogeneous data and are extracted in decreasing order of importance. The first principle component accounts for as much as possible of the variation in the heterogeneous data. The second component accounts for as much of the remaining variability being uncorrelated with the first component. The objective of this type of analysis is to see whether the first few components account for most of the variation in the original data. This helps to identify the most important directions of variability in a large set of heterogeneous dataset (Destefanis, Barge, Brugiapaglia, & Tassone, 2000; Šnirc et al., 2017). The correlation between a component and a variable estimates the information they share. In PCA, this correlation is called a loading (Destefanis et al., 2000). PC loading shows the relationship between the originally measured variables and the extracted PC (Abdi & Williams, 2010). Thus, PCA is a very effective procedure for obtaining synthetic judgment of meat quality, through the reduction in dimensionality, which permits visual interpretation of the data represented by two‐dimensional scatter plots (Destefanis et al., 2000; Šnirc et al., 2017). These scatter plots are called PC loading plots. In the loading plots, variables close together are positively correlated, while those lying opposite to each other tend to have negative correlation. The more a variable is away from the axis origin, the more it loads onto that PC (Baardseth, Helgesen, & Isaksson, 1996). Various studies have employed PCA to assess meat and meat products quality characteristics (Boyacı et al., 2014; Cañeque et al., 2004; Destefanis et al., 2000; Karlsson, 1992; Liu, Lyon, Windham, Lyon, & Savage, 2004). It is therefore an ideal tool for studying quality characteristics in beef rounds extended with gum arabic for which such analysis is yet to be reported.

Therefore, the aim of this work was to study relationships among the various physicochemical and sensory characteristics and allow the grouping of samples with similar characteristics in beef rounds injected with brines containing gum arabic from *Acacia senegal* var*. kerensis* at different levels. This article reports the results of the PCA for 24 beef round quality measurements (physicochemical and sensory attributes) measured.

## 2 MATERIALS AND METHODS

### 2.1 Sample preparation

This research was carried out at the Castle Meat Products factory and the Egerton University, Nakuru, Kenya. The beef rounds were injected with brines containing gum arabic from *A. senegal var. kerensis*. Beef injection brine contained standard recommended amounts of sodium chloride (2%), sodium nitrate (0.02%), sodium tripolyphosphate (0.5%), and sodium ascorbate (0.0547%) with different levels of gum arabic (1%, 1.5%, 2%, 2.5%, and 3%). For comparison, curing brine solutions containing SPC (3.5%) were used. Meat cuts weighing 3.5 kg consisting of pieces from the beef round were trimmed of external fat, skin, membranes, and the silver skin, and injected with curing brines using a manual injector pump (Friedr. DICK Hand Brine Injector Pump) followed by massaging for 3 hr to evenly distribute the brine. Rounds were then kept for 18 hr at 4°C after which they were cooked and sliced uniformly. A total of 24 variables were analyzed on the cooked beef round samples.

### 2.2 Cook yield and expressible moisture

The centrifugation method was followed in the determination of expressible moisture using a centrifuge DSC‐200A (Aron Laboratory Instruments, Taiwan). This was achieved by centrifuging 10 g sample at 860*g* for 7.5 min at 20°C (Zhang, Mittal, & Barbut, 1995). Cook yield was determined as the percentage of cooked sample weight over the raw weight of the noninjected sample.

### 2.3 Possible extension level and gum level

Possible injection level was taken as the percent increase in weight of the injected beef after 18 hr storage at 4°C, while the actual gum arabic in injected beef rounds was calculated based on the possible injection level achieved for each extended beef cut.

### 2.4 Moisture content and protein content

The moisture was determined by the AOAC method 950.46, while the crude protein by AOAC method 981.10 (AOAC, 2000).

### 2.5 Sensory analysis

Sensory evaluation was performed by a trained descriptive attribute sensory panel consisting of seven persons. This panel was selected and trained according to the procedures of Meilgaard, Carr, and Civille (2006) on sensory profiling. Cooked beef samples were evaluated for flavor (beefy, beef fat, livery), feeling factors (astringent, chemical burn, chemical taste), texture (springiness, juiciness, hardness, and denseness), and basic tastes (saltiness and sourness) using the 16‐point spectrum universal intensity scale where 0 = *absence of attribute* and 15 = *extremely intense*. In addition, cooked beef round samples were evaluated for color as follows: overall color using an 8‐point descriptive scale (1 = *gray*, 8 = *dark reddish pink*), percent surface discoloration (gray and/or brown) (1 = *no surface gray color 0%, no surface brown color 0%*; 6 = *total surface gray color 100%, total surface brown color 100%*), and iridescence (1 = *none 0%*, 6 = *very strong 100%*) using a 6‐point descriptive scale for both. Definitions of sensory terms as discussed and agreed on by the panelists are shown in Table 1. Overall acceptability was done using a 5‐point hedonic scale.

**Table 1:** Descriptions of the full range of sensory attributes as used in the descriptive sensory analyses

Characteristic	Attribute	Definition
Flavor^a^	Beefy (B)	Flavors and aromatics associated with boiled meat
	Beef fat (BF)	Sensation caused by various levels of fat in the beef
	Livery (L)	Taste found in animal organs
Texture^a^	Hardness (H)	Force required to bite through sample
	Denseness (DE)	Compactness of the cross section
	Springiness (SP)	Degree to which sample returns to original shape after a certain time period
	Juiciness (J)	Sensation caused by meats with higher levels of juices
Basic tastes^a^	Saltiness (ST)	Basic taste stimulated by sodium salts
	Soured (S)	Basic taste stimulated by acids
Feeling factors^a^	Astringent (A)	Mouth‐drying and harsh sensation
	Chemical taste (CT)	Taste associated with compounds such as cleaning detergents
	Chemical burn (CB)	Chemical feeling factor associated with irritating substances (to the mucous membrane of the oral cavity)
	Metallic taste (MT)	Taste associated with various metal flavors that could be found in meat
Color	Overall color intensity (CI)	Color of cured meat on the surface and the inside (1 = gray, 8 = dark reddish pink)
	Gray color (GC)	Color of meat on the surface and the inside (1 = no surface gray color 0%, 6 = total surface gray color 100%)
	Brown color (BC)	Color of meat on the surface and the inside (1 = no surface brown color 0%, 6 = total surface brown color 100%)
	Iridescence (I)	The property of meat surfaces appearing to change color as the angle of view changes. (1 = none 0%, 6 = very strong 100%).

a Accessed using a 16‐point spectrum universal intensity scale where 0 = absence of attribute and 15 = extremely intense.

### 2.6 Statistical analysis

This study employed a completely randomized design in a factorial arrangement with two factors (binder levels [gum arabic and SPC] and injection levels). Statistical analysis was done using The Unscrambler X 10.4 software for PCA to study attribute sample relationships so as to reduce the set of beef round quality attributes (24 variables) to a number of linearly uncorrelated variables, and for the determination of linear correlation coefficients at *p* = .05 level of significance.

## 3 RESULTS AND DISCUSSION

The means and standard deviations of the variables determined are shown in Table 2. The coefficients of variation were lower than 5% for moisture and cook yield at 2.27% and 2.33%, respectively. All other measurements had coefficients of variation lower than 30% other than livery, soured, gray color, chemical burn, expressible moisture, gum arabic in beef round, and chemical taste. The highest coefficient of variation was recorded for chemical taste at 60.61%.

**Table 2:** Mean, *SD*, and coefficient of variations (CV) of the beef round quality measurements

Characteristics	Attribute	Mean ± *SD*	CV
Flavor	Beefy	8.91 ± 1.23	13.76
	Beef fat	5.43 ± 0.87	16.05
	Livery	4.27 ± 1.20	28.11
Texture	Hardness	7.23 ± 1.79	24.75
	Denseness	8.77 ± 1.19	13.52
	Springiness	6.71 ± 1.41	21.03
	Juiciness	8.01 ± 1.46	18.21
Basic tastes	Saltiness	5.58 ± 1.08	19.43
	Soured	2.94 ± 0.91	31.11
Feeling factors	Astringent	4.97 ± 1.28	25.84
	Chemical taste	0.89 ± 0.54	60.61
	Chemical burn	1.07 ± 0.36	33.39
	Metallic taste	1.10 ± 0.27	24.61
Color	Color intensity	5.64 ± 0.83	14.67
	Gray color	2.52 ± 0.79	31.20
	Brown color	3.29 ± 0.49	14.92
	Iridescence	2.66 ± 0.42	15.74
General liking	Acceptability	3.32 ± 0.45	13.62
Physicochemical tests	Expressible moisture (%)	14.56 ± 4.89	33.56
	Moisture (%)	71.72 ± 1.63	2.27
	Cook yield (%)	87.55 ± 2.04	2.33
	Possible injection (%)	31.67 ± 3.1	9.79
	Gum arabic in round (%)	1.96 ± 0.74	37.62
	Protein (%)	22.41 ± 2.29	10.22

**Figure 1:**
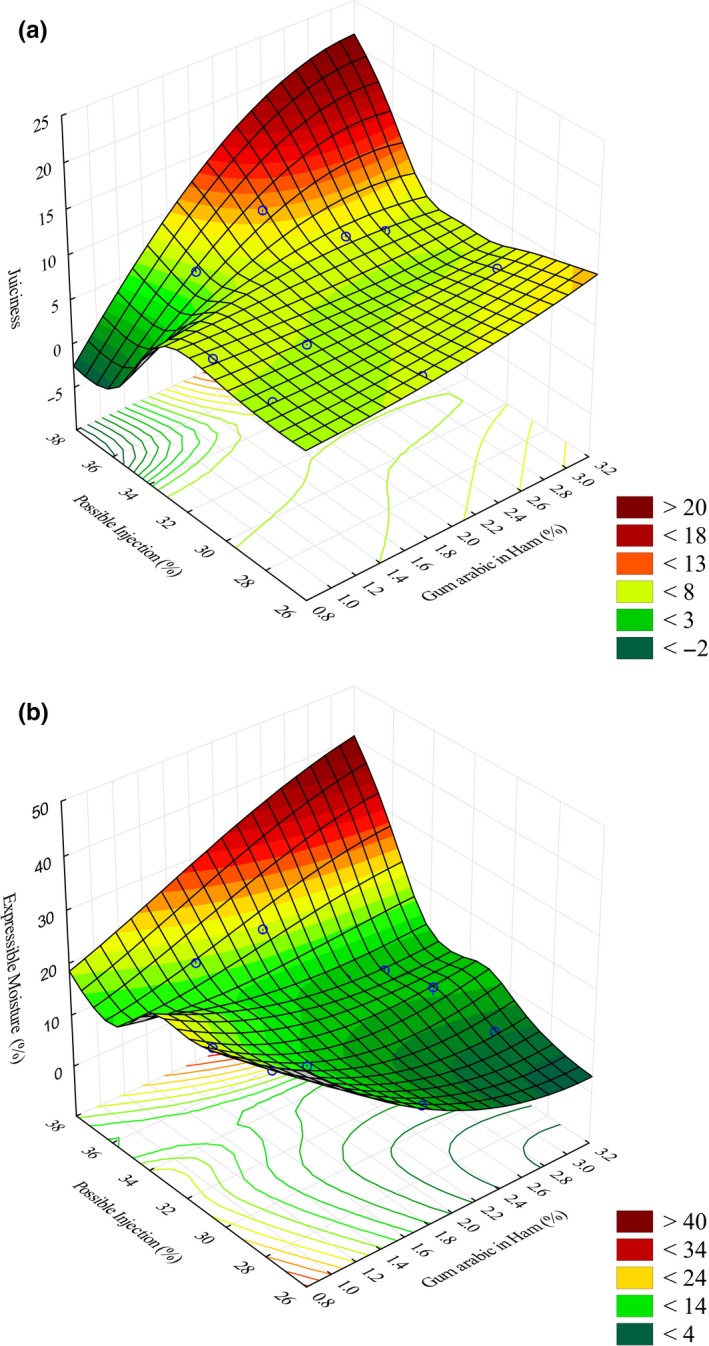
3D surface plot for (a) achievable gum arabic in beef round (%) versus possible injection (%) versus juiciness, and (b) achievable gum arabic level in beef round (%) versus possible injection (%) versus expressible moisture (%)

Table 3 shows the correlation coefficients between the quality attributes tested. Various attributes were found to be highly correlated with each other. The highest significant negative and positive correlations were recorded between color intensity and gray color and between color intensity and brown color, respectively. This was expected since low‐cured color intensity usually results in loss of brown color in the cooked product. Similarly, samples with high‐cured color intensity would have higher brown color rating. Color intensity was also negatively correlated to beefy, livery, and sourness attributes, while gray color was positively correlated to these attributes. This is unlike what was reported by Liu et al. (2004). In their study, none of the color attributes was correlated with any of the other sensory attributes. Protein content was positively correlated with juiciness as well as overall acceptability. Gum arabic level was positively correlated to expressible moisture. In addition, cook yield and overall acceptability were positively correlated to juiciness. Similar results were reported by Destefanis et al. (2000). In their study, Destefanis et al. (2000) found overall acceptability and juiciness to be negatively correlated to cooking losses. Expressible moisture was positively correlated to juiciness, but negatively correlated to the gum level, indicating that increasing gum level resulted in a decrease in expressible moisture; meaning that there was an improvement in water‐holding capacity in the beef rounds with increased gum level as shown in Figure 1a,b as reported earlier (Mwove et al., 2016).

**Table 3:** Correlation coefficients between the beef rounds quality measurements

	B	BF	L	AS	CT	CB	MT	H	DS	SP	J	ST	SR	CI	GC	BC	I	OA	EM	MC	CY	PI	GH
BF	0.16																						
L	0.36^a^	0.36^a^																					
AS	−0.10	−0.23	−0.42^a^																				
CT	−0.19	0.24	0.44^a^	0.09																			
CB	−0.19	−0.17	−0.08	0.02	0.47^a^																		
MT	0.30	0.39^a^	−0.05	0.25	0.14	−0.29																	
H	−0.21	0.11	−0.43^a^	0.45^a^	−0.38^a^	−0.57^a^	0.20																
DS	−0.23	0.01	−0.32	0.46^a^	−0.40^a^	−0.79^a^	0.27	0.79^a^															
SP	0.11	0.30	−0.32	0.40^a^	−0.27	−0.64^a^	0.59^a^	0.84^a^	0.72^a^														
J	0.14	0.38^a^	0.55^a^	−0.21	0.43^a^	−0.29	0.46^a^	−0.33	0.07	−0.05													
ST	−0.07	0.19	0.33	0.00	0.01	0.04	−0.15	−0.26	0.02	−0.45^a^	0.31												
SR	0.33	0.24	0.58^a^	−0.08	0.43^a^	0.30	−0.05	−0.55^a^	−0.41^a^	−0.35	0.19	0.27											
CI	−0.42^a^	−0.10	−0.45^a^	0.13	−0.13	0.39^a^	−0.30	0.24	−0.07	−0.09	−0.38^a^	0.11	−0.41^a^										
GC	0.43^a^	0.18	0.58^a^	−0.33	−0.05	−0.48^a^	0.08	−0.14	0.07	0.05	0.23	0.01	0.40^a^	−0.90^a^									
BC	−0.13	0.14	−0.13	−0.03	−0.01	0.34	−0.15	0.17	−0.20	−0.01	−0.21	0.04	−0.14	0.88^a^	−0.75^a^								
I	0.54^a^	0.53^a^	0.48^a^	−0.01	0.16	−0.52^a^	0.57^a^	0.16	0.24	0.51^a^	0.62^a^	−0.16	0.07	−0.43^a^	0.36^a^	−0.11							
OA	−0.15	0.32	0.51^a^	−0.19	0.48^a^	0.04	0.07	−0.47^a^	−0.12	−0.47^a^	0.76^a^	0.70^a^	0.19	−0.18	0.13	−0.23	0.16						
EM	0.02	−0.13	0.24	−0.37^a^	0.63^a^	0.54^a^	−0.06	−0.69^a^	−0.73^a^	−0.50^a^	0.21	−0.38^a^	0.22	−0.21	0.04	−0.15	−0.04	0.19					
MC	−0.12	−0.15	−0.02	0.45^a^	0.19	−0.11	−0.18	0.35	0.26	0.29	−0.31	−0.39^a^	0.00	−0.36^a^	0.31	−0.46^a^	0.11	−0.24	0.03				
CY	0.09	0.32	0.63^a^	−0.15	0.03	−0.53^a^	−0.09	0.10	0.32	0.03	0.51^a^	0.42^a^	−0.02	−0.31	0.46^a^	−0.24	0.55^a^	0.55^a^	−0.27	0.20			
PI	−0.07	0.01	0.29	0.16	0.45^a^	0.35	0.22	−0.32	−0.25	−0.31	0.29	0.34	−0.01	−0.12	0.00	−0.21	0.08	0.56^a^	0.22	0.06	0.27		
GH	0.05	0.44^a^	0.37^a^	0.12	−0.51^a^	−0.64^a^	0.26	0.54^a^	0.65^a^	0.46^a^	0.20	0.53^a^	−0.15	−0.25	0.46^a^	−0.26	0.34	0.26	−0.81^a^	0.03	0.79^a^	0.15	
PC	0.11	0.47^a^	−0.24	−0.07	−0.08	0.01	0.52^a^	0.09	0.03	0.36^a^	0.27	−0.16	−0.27	0.28	−0.41^a^	0.39^a^	0.42^a^	−0.01	−0.05	−0.43^a^	−0.09	0.04	0.11

B, beefy; BF, beef fat; L, livery; H, hardness; DE, denseness; SP, springiness; J, juiciness; ST, saltiness; S, soured; A, astringent; CT, chemical taste; CB, chemical burn; MT, metallic taste; CI, color intensity; GC, gray color; BC, brown color; I, iridescence; OA, overall acceptability; EM, expressible moisture (%); MC, moisture content (%); CY, cook yield (%); PI, possible injection (%); GA, gum arabic in round (%); PC, protein content (%).

a Absolute value >0.35 was objectively considered to have significant correlation, and a value between 0.20 and 0.35 had moderate (Liu et al., 2004).

In PCA, the first seven PCs were extracted explaining over 95% of the total variance for the beef round quality parameters (Table 4). The first three of these PCs accounted for 73.6% of the variance observed in the 24 beef round quality parameters (PC1 = 45.3%, PC2 = 17.3%, and PC3 = 11.0%). Cañeque et al. (2004) identified eight PCs explaining 74% of variation in meat quality measurements including moisture, water‐holding capacity, cooking losses, juiciness, hardness, springiness, flavor, and overall acceptability, while Liu et al. (2004) extracted seven PCs explaining 69.2% of the total variation for attributes including physical, color, and sensory characteristics of chicken breasts.

**Table 4:** Explained variance

	Eigenvalue	Explained variance	Cumulative %
PC1	10.88	45.34	45.34
PC2	4.16	17.32	62.66
PC3	2.63	10.97	73.63
PC4	2.16	8.98	82.62
PC5	1.76	7.35	89.97
PC6	0.79	3.31	93.28
PC7	0.54	2.25	95.52

The principal component loadings for beef round quality measurements are shown in Table 5. The first PC was characterized by texture attributes (hardness and denseness), feeling attributes (chemical taste and chemical burn), and two physicochemical properties (expressible moisture and achieved gum arabic in beef round). The second PC was defined by taste attribute (saltiness), physicochemical attributes (cook yield and possible injection), and overall acceptability, while the third PC was characterized by metallic feeling factor, color attributes (gray color and brown color), and physicochemical attributes (moisture and protein content).

**Table 5:** Correlation loadings for 24 beef round quality attributes

	PC1	PC2	PC3	PC4	PC5	PC6	PC7
Beefy	0.045	0.017	0.083	0.403	0.106	0.799	0.154
Beef fat	−0.134	0.207	0.406	0.408	0.297	0.101	0.080
Livery	0.313	0.498	−0.250	0.641	0.115	0.132	0.087
Hardness	−0.781	−0.172	−0.023	−0.304	0.423	−0.158	0.062
Denseness	−0.796	0.041	−0.076	−0.067	0.388	−0.207	−0.331
Springiness	−0.609	−0.189	0.220	−0.168	0.660	0.175	−0.081
Juiciness	0.246	0.478	0.295	0.530	0.277	−0.124	−0.491
Saltiness	−0.237	0.611	−0.054	0.306	−0.595	−0.103	−0.205
Soured	0.286	0.059	−0.258	0.398	−0.206	0.521	−0.259
Astringent	−0.383	0.116	−0.121	−0.659	0.113	0.239	−0.446
Chemical taste	0.647	0.283	−0.055	−0.102	0.210	−0.073	−0.344
Chemical burn	0.604	−0.030	0.111	−0.409	−0.502	0.066	0.113
Metallic taste	−0.082	0.187	0.532	−0.094	0.392	0.394	−0.412
Color intensity	−0.218	−0.221	0.367	−0.322	−0.444	−0.447	0.174
Gray color	0.051	0.211	−0.502	0.497	0.329	0.366	0.094
Brown color	−0.153	−0.245	0.467	−0.044	−0.321	−0.225	0.207
Iridescence	−0.070	0.299	0.281	0.398	0.726	0.281	−0.041
Acceptability	0.285	0.708	0.064	0.341	−0.107	−0.311	−0.310
Expressible moisture (%)	0.984	−0.125	0.002	−0.017	0.115	−0.036	−0.004
Moisture (%)	−0.036	0.035	−0.627	−0.394	0.586	0.080	0.102
Cook yield (%)	−0.227	0.672	−0.208	0.477	0.348	−0.231	0.147
Possible injection (%)	0.340	0.858	0.090	−0.360	−0.053	0.051	0.043
Gum arabic in round (%)	−0.773	0.592	0.020	0.328	0.125	0.059	0.073
Protein (%)	−0.073	−0.003	0.961	0.006	0.199	0.030	0.071

**Figure 2:**
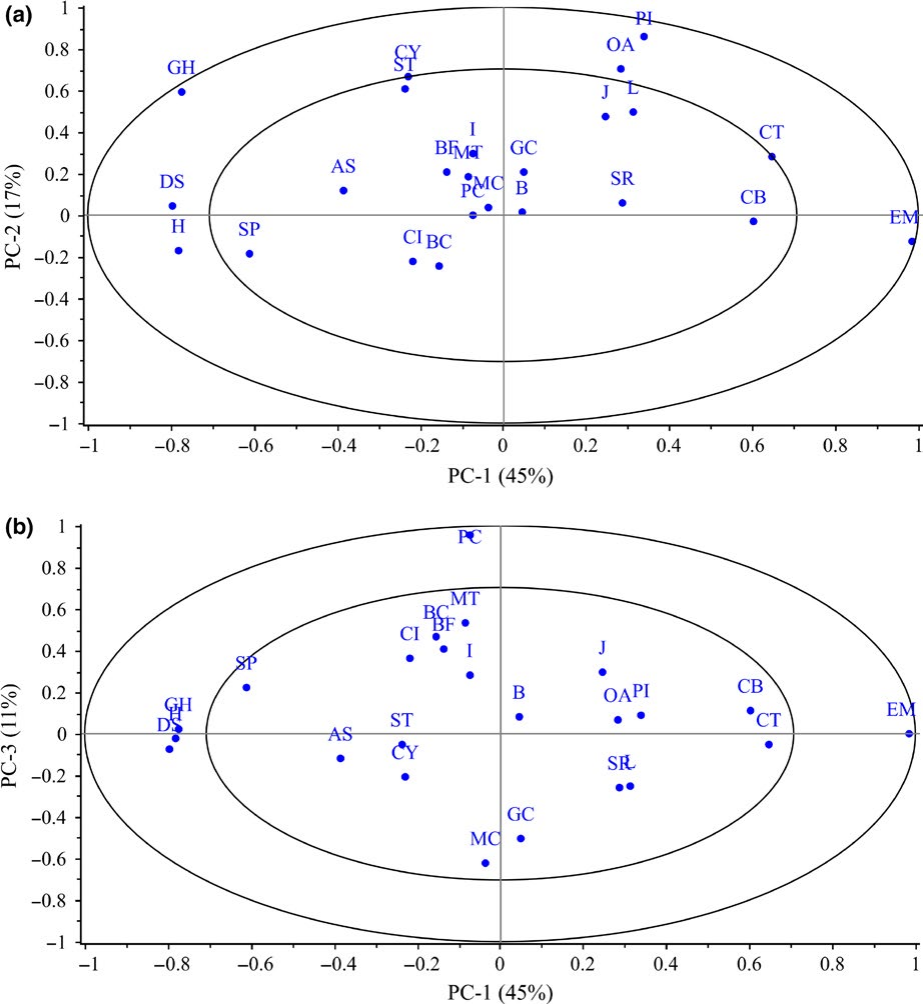
Biplot for (a) factor 1 and factor 2, and (b) factor 1 and factor 3. B, beefy; BF, beef fat; L, livery; H, hardness; DE, denseness; SP, springiness; J, juiciness; ST, saltiness; S, soured; A, astringent; CT, chemical taste; CB, chemical burn; MT, metallic taste; CI, color intensity; GC, gray color; BC, brown color; I, iridescence; OA, overall acceptability; EM, expressible moisture (%); MC, moisture content (%); CY, cook yield (%); PI, possible injection (%); GA, gum arabic in round (%); PC, protein content (%)

Figure 2a,b shows the correlation loading plots of the measurements of beef round quality on the first two PCs. The measurements and PCs are interpreted according to the correlations between each parameters and each PC, thus measurements close to each other are positively correlated, measurements separated 180° are negatively correlated, whereas if they are separated by 90°, they are independent (Cañeque et al., 2004). In Figure 2a, hardness, springiness, and achieved gum arabic in beef round are placed far from the first PC indicating their importance in defining this PC. Their negative loading shows that their decrease would result in an increase in this PC. Similarly, expressible moisture is far from this PC with a positive loading indicating that its increase would result in a decrease in this PC. In addition, hardness and springiness are located close to one another indicating that they are positively correlated with each, other but negatively correlated to juiciness which is located on the opposite quadrant. Possible injection and juiciness were located close to overall acceptability, showing that these two may be very important in defining the acceptability of the products. In this work, samples that were highly desired were also rated highly for juiciness. In addition, samples that had higher possible injection also had higher juiciness (Mwove et al., 2016).

Overall acceptability, possible injection, and cook yield were highly positively correlated with the second PC. As reported earlier in Table 3, these three were positively correlated with each other. This makes possible injection and cook yield very crucial in defining the quality of beef rounds containing gum arabic.

In Figure 2b, color intensity and brown color are placed on opposite quadrants to the gray color showing that these were negatively correlated. They are however placed closer to the first PC showing their less importance in defining it. Nevertheless, in respect to the third PC, gray color and brown color together with moisture and protein content are located far away, and hence their usefulness in defining the third PC.

**Figure 3:**
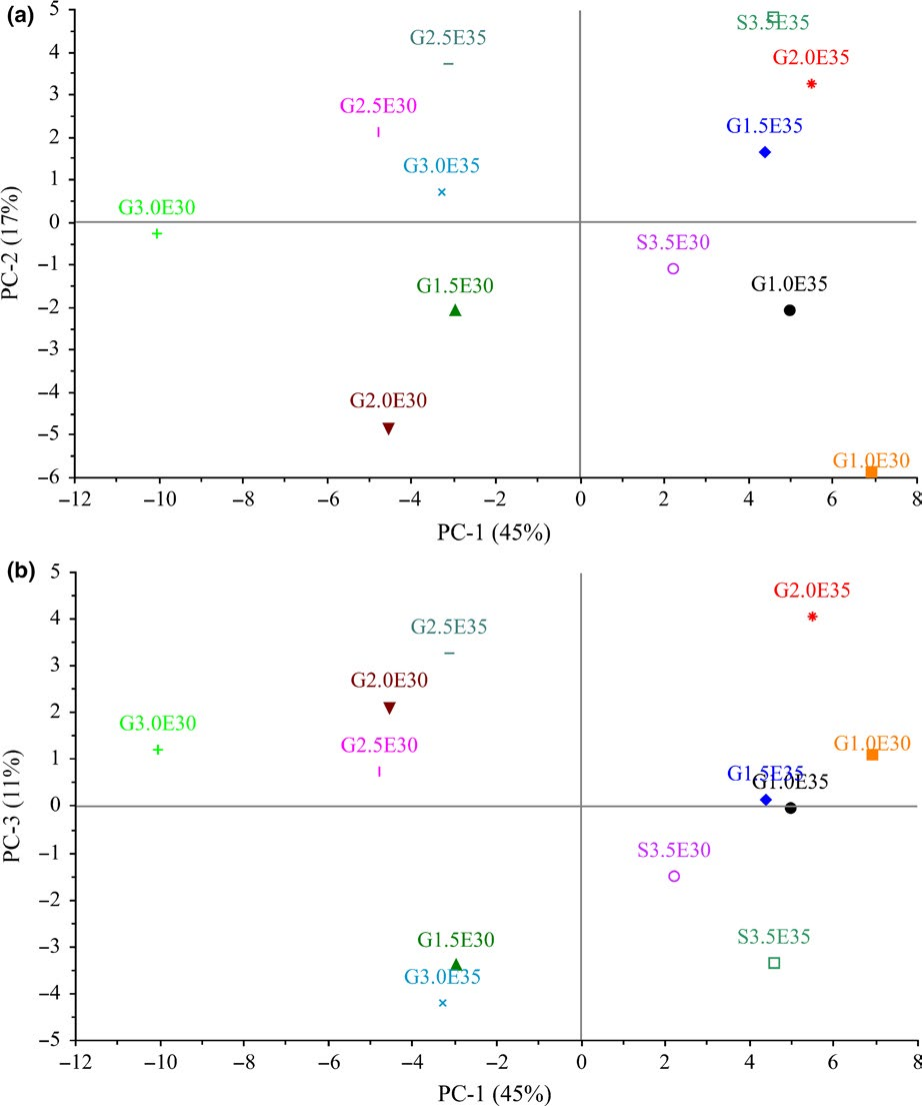
Scores biplot for (a) PC1 versus PC2, and (b) PC1 versus PC3. G1.0E30, G1.5E30, G2.0E30, G2.5E30, G3.0E30, G1.0E35, G1.5E35, G2E35, G2.5E35, and G3E35 represent 1%, 1.5%, 2%, 2.5%, and 3% gum arabic injected beef rounds at 30% (E30) and 35% (E35) injection levels, while SPC30 and SPC35 are the 3.5% SPC‐injected beef rounds at 30% and 35% injection levels, respectively

Figure 3a,b shows the scores biplot for PC1 versus PC2 and PC1 versus PC3, respectively. The score plot shows the location of the objects in the multivariate space of two principal component score vectors (Destefanis et al., 2000). Two groups of samples were clearly separated as seen in Figure 3a. Samples extended to 35% level were placed on the upper side of the biplot where most sensory attributes as well as physicochemical attributes were placed except hardness, springiness, color intensity, brown color, and expressible moisture. Hardness and springiness were earlier reported to be lower in samples extended to 35% level as compared to those extended to 30% (Mwove et al., 2016). However, these samples were high in expressible moisture which is positively loading on PC1 (Table 5). In Figure 3b, samples extended to 30% are located on the left side, while those extended to 35% are located on the right side near juiciness, overall acceptability, and possible injection (Figure 2b). This shows that samples injected to 35% level were juicier and had the highest overall acceptability rating (Mwove et al., 2016).

Beef samples extended with gum arabic were also displayed on the left side, while those containing SPC were placed on the right side (Figure 3a). In Figure 3b, SPC containing samples are located on their own at the bottom right‐side quadrant where sourness and gray color attributes are. This indicates that samples containing SPC were higher in these attributes as compared to the gum arabic extended beef round samples.

## 4 CONCLUSION

The results of PCA showed that the physicochemical and sensory attributes of beef hams extended with gum arabic and SPC were highly correlated. Several significant correlations were found between beef round quality parameters. The highest significant negative and positive correlations were recorded between color intensity and gray color and between color intensity and brown color, respectively. PCA revealed that texture characteristics (hardness, denseness) as well as expressible moisture and achieved gum arabic in beef round were important in defining PC1. In addition, the distribution of the samples on the axes of the first two PCs allowed for differentiation of samples injected to 30% injection level which were placed on the upper side of the biplot from those injected to 35% which were placed on the lower side. Differentiation of beef samples extended with gum arabic and those containing SPC were also visible when scores for the first and third PCs were plotted. Thus, it was possible to discriminate groups of samples based on types and levels of binders used as well as the levels of injection indicating differences in beef round characteristics. Thus, PCA was very efficient in analyzing the physicochemical and sensory characteristics of beef rounds extended with gum arabic.

## CONFLICT OF INTEREST

The authors declare that they do not have any conflict of interest.
